# Epigenetic Control of a Local Chromatin Landscape

**DOI:** 10.3390/ijms21030943

**Published:** 2020-01-31

**Authors:** Anna M. Chiarella, Dongbo Lu, Nathaniel A. Hathaway

**Affiliations:** Division of Chemical Biology and Medicinal Chemistry, Center for Integrative Chemical Biology and Drug Discovery, UNC Eshelman School of Pharmacy, Chapel Hill, NC 27514, USA; ac4577@cumc.columbia.edu (A.M.C.); dongbo@email.unc.edu (D.L.)

**Keywords:** chromatin, CRISPR, dose-dependent, gene-specific, epigenomics, epigenetics

## Abstract

Proper regulation of the chromatin landscape is essential for maintaining eukaryotic cell identity and diverse cellular processes. The importance of the epigenome comes, in part, from the ability to influence gene expression through patterns in DNA methylation, histone tail modification, and chromatin architecture. Decades of research have associated this process of chromatin regulation and gene expression with human diseased states. With the goal of understanding how chromatin dysregulation contributes to disease, as well as preventing or reversing this type of dysregulation, a multidisciplinary effort has been launched to control the epigenome. Chemicals that alter the epigenome have been used in labs and in clinics since the 1970s, but more recently there has been a shift in this effort towards manipulating the chromatin landscape in a locus-specific manner. This review will provide an overview of chromatin biology to set the stage for the type of control being discussed, evaluate the recent technological advances made in controlling specific regions of chromatin, and consider the translational applications of these works.

## 1. Introduction

### 1.1. Chromatin

Differential regulation of the chromatin landscape (composed of DNA wrapped around histone linkers or octamers) allows for genetically identical eukaryotic cells to perform specific, discrete functions [1]. This is achieved by regulating different gene expression profiles, which in turn result in a cell adopting specific proteomes to conduct specialized functions in a multicellular organism. A nucleosome represents the core building block of chromatin and is comprised of 147 DNA base pairs (bp) wrapped around histone protein octamers (two sets of each histone protein H2A, H2B, H3, and H4) [2]. For three billion base pairs of DNA to fit within a 10 μm nucleus while still allowing functional information to be expressed, chromatin needs to be folded in a precise, organized manner [3]. Chromatin is differentially compacted along a spectrum of condensation states, with the term “heterochromatin” referring to areas of tight compaction (associated with low transcriptional activity) and “euchromatin” referring to areas of less compaction (associated with high transcriptional activity) [4].

Chromatin compaction has been identified as a physical determinant of DNA’s accessibility to transcription-initiating proteins, and these processes are highly regulated through deposition of histone protein variants, ATP-dependent nucleosome remodeling, methylation of DNA, and posttranslational modifications of histone tails [5]. These interrelated processes work in concert to control transcriptional activity, DNA replication, damage repair, and nuclear organization [4,6,7,8]. For the purposes of this review, we focus primarily on the contribution of DNA methylation and histone tail modifications to the regulation of gene expression.

Protruding from nucleosomes are N-terminal histone tails, capable of being post- translationally chemically modified, and DNA bases can also be modified at cytosine (C) and adenine (A) nucleotides [9,10,11,12]. Specific combinations of histone and DNA modifications give rise to an epigenetic code, with specific modifications being associated with high or low levels of gene expression [13]. To date, four nucleotide modifications have been observed: methylation, hydroxy-methylation, formylation, and carboxylation [14]. Over ten modifications have been identified for histone tails, with more modifications currently being validated and characterized [15,16].

The Allis group coined the phrase “the histone code” to describe the observation that distinct histone marks function sequentially or in combination to initiate distinct downstream changes [11,13]. Changing modifications of nucleotides or histone tails requires a series of proteins: writers, erasers, and readers. “Writer” enzymes deposit the nucleotide or histone tail modification, “eraser” enzymes remove the modification, and “reader” domains recognize a modification, initiating downstream consequences such as recruitment of transcription factors [5]. Two of the most widely studied nucleotide modifications are methylation (of DNA and of the lysine residue) and acetylation (of the lysine residue) [17]. These chromatin-based mechanisms are commonly used in the field of epigenetic biotechnology to control gene expression. Importantly, there is debate between scientists about correlation versus causation. Are the actual histone marks and DNA modifications important for changes in transcription, or are the modifications passengers? There are examples of evidence for both models [18,19,20]. Fortunately, gene-specific manipulation of chromatin can serve as a new tool to further study these models. The classification and function of the proteins mediating DNA methylation and lysine methylation/acetylation are important for the design of chromatin recruitment tools.

### 1.2. DNA Methylation

Eukaryotic DNA methylation is most often observed as 5-methylcytosine in the context of a cytosine–guanine (CpG) dinucleotide and is strongly associated with gene repression, though there are some notable exceptions [21,22]. The mark is deposited by DNA methyltransferases (DNMTs), enzymes that transfer a methyl group from S-adenosyl-L-methionine (SAM) to cytosine at the 5′ position [23]. Within the mammalian DNMT family, DNMT1 is considered the maintenance methyltransferase, as it propagates the methylation pattern from parental to daughter strands during cell replication [24,25]. DNMT3A and DNMT3B are both considered the de novo methyltransferases, as they methylate both hemi-methylated and un-methylated DNA. DNMT3A/B are critical for the initiation of DNA methylation during gametogenesis and the early stages of embryogenesis [21,26]. Importantly, these are the DNMT family members that have been used for epigenomic manipulation (discussed in Section 3 of this review), in part due to the stability of DNA methylation to progress through cell replication [27,28]. The DNA methylation “erasers” are part of the ten-eleven translocation (TET) family. It has been shown that this family of enzymes catalyze the stepwise oxidation of methyl groups, ultimately removing the methylation from the cytosine [14] in concert with the TDG DNA glycosylase [29]. The proteins that recognize methylated DNA are the methyl CpG binding proteins (MBPs), including zinc finger (ZnF) domain-, SRA domain-, and methyl CpG binding domain- (MBD-) containing proteins [30]. Following recognition of methylated DNA, compaction and transcriptional silencing is initiated, in part through coordinated recruitment of chromatin remodeling complexes.

### 1.3. Histone Lysine Methylation

The lysine residues on histone tails can be mono-, di-, or tri-methylated by a set of histone writer proteins, generically referred to as histone lysine methyltransferases (KMTs). Methyl groups are transferred from the methyl donor, adenosyl-methionine. KMTs are split into two families based on the presence (or lack) of a SET domain [17]. Mammalian examples of SET-containing enzymes include H3K4 methyltransferases (such as SET1A/B and SET7 [31,32]), H3K9 methyltransferases (such as EHMT2/G9a and SUV39H1/2) [6,33,34,35], H3K27 methyltransferases (such as EZH1/2) [36,37,38], H3K36 methyltransferases (such as NSD1) [39], and H4K20 methyltransferases (such as SUV4-20H1/H2) [40]. Fewer studies have been done on KMTs lacking the SET domain other than the highly studied H3K79 methyltransferase, DOT1 [41,42,43]. Proteins that recognize histone lysine methylation contain one, or multiple, reader domains: chromodomain [44], chromobarrel domain [45], double chromodomain [46], PWWP domain [47], plant homeodomain [48], WD40 domain [49], malignant brain tumor domain [50], and Tudor domain [17,51]. Some of these reader domains have been discovered in proteins alongside other reader domains (i.e., UHRF1 recognizes hemi-methylated DNA and H3K9me^3^) [52]. The removal of lysine methylation is performed by one of two ways: lysine-specific demethylase (LSD) members, LSD1/KDM1A and LSD2/KDM1B, contain an amine oxidase (AOD) domain responsible for demethylase activity in a flavin adenine dinucleotide (FAD)-dependent manner [53], whereas Jumonji C (JmjC) domain-containing enzymes [54] utilize the co-substrates 2-oxoglutarate (2OG) and dioxygen with cofactor Fe(II) [55,56,57,58,59]. Unlike DNA methylation, the transcriptional influence of lysine methylation is strongly context dependent [60]. For example, H3K36me^3^ is observed in the gene body of transcriptionally active genes, whereas H3K9me^3^ is linked with gene repression when concentrated near transcriptional start sites (TSS) [60,61]. For this reason, the chromodomain, a reader of H3K9me^3^, has been used for recruitment to induce targeted control of chromatin (in systems discussed in Section 3 of this review).

### 1.4. Histone Lysine Acetylation 

Lysine residues can be acetylated, which typically results in a less condensed chromatin state coupled with an increase in transcriptional activity [62,63]. Histone acetyl transferases (HATs) are the writer enzymes that deposit acetylation marks from acetyl-CoA. HAT families are classified based on cellular location, with Type A HATs functioning on chromatin in the nucleus and Type B HATs acting on newly translated histones in the cytoplasm. The Type A HATs are further classified into five subtypes based on their structural and functional characteristics: GNAT, MYST, p300/CREB, TAF250, and SRC/NCoA [17]. The most commonly used HAT family in the field of epigenetic biotechnology is the p300/CBP family of HATs, KAT3A/CBP and KAT3B/p300, which acetylates all four histone subunits and contains several domains including a KIX domain, a bromodomain, and a HAT domain [64]. Histone deacetylases (HDACs) are the eraser enzymes that remove lysine acetylation, supporting a transcriptionally silenced state. This set of enzymes is subdivided into Class I, IIa, IIb, III, and IV [17,65,66]. HDAC Class I, IIa, IIb, and IV require an interaction with zinc, whereas class III, or sirtuin, requires an HDACs function with NAD^+^ [17,65,67]. For chromatin manipulation with biotechnology, class I HDACs (HDAC1/2/3/8) are most commonly used, in part because all are observed predominantly in the nucleus and are expressed in most tissue types. In addition, HDAC1/2/3 function in collaboration with repressive complexes, which is important for downstream recruitment and targeted transcriptional repression. The three peptide folds that interact with histone lysine acetylation are the bromodomain (BRD), double PHD finger domain, and Yeats domain [17]. BRD-containing proteins are the most abundant lysine acetylation readers and are sub-classified into eight families based on similarities in structure and sequence [17,68,69] including the BRD II family comprising the Bromodomain and Extra-Terminal Domain (BET) proteins BRD2/3/4 and BRDT. BET proteins recognize H4K12ac, H3K14 ac, H4K5ac, and H4K8ac. Lastly, BRD family III domains are found in many proteins that are ubiquitously expressed across cell types including EP300 and CREBBP [69].

### 1.5. Chromatin and Transcription

Models that describe the close interplay between specific chromatin environments and their associated transcriptional activities are based on two characteristics: the physical accessibility of the DNA due to the level of chromatin compaction and the recruitment of transcriptional machinery by chromatin modifications. At the eukaryotic promoter, RNA polymerase II (Pol II) binds to DNA, in coordination with other general transcription factors, and forms the preinitiation complex (PIC) [70]. The chromatin environment surrounding the promoter, as well as functionally relevant enhancer regions, is important for Pol II binding, PIC formation, subsequent elongation, and proper termination. For example, variations in nucleosome acetylation levels impact how readily the transcriptional machinery runs along the DNA [71]. Additionally, H4K16ac blocks chromatin compaction and increases overall accessibility of the DNA to the transcriptional machinery [72]. Recruitment of SetD2 by Pol II is an import step that occurs during transcriptional elongation. Upon Set2 recruitment, H3K36me^3^ is deposited, followed by hypoacetylation of the gene body [73]. Lastly, prolonged recruitment of HP1 (through recognition of H3K9me^3^) results in the deposition of DNA methylation, an epigenetically stable and transcriptionally repressive modification [74]. While this interplay is more complicated than what has been described above, these examples illustrate how transcriptional regulation and the chromatin environment are related. Given this tight interaction, manipulating the function, recruitment, and/or expression of chromatin modifying proteins can subsequently control the expression of essential genes [75].

## 2. Global Control of Chromatin

For decades, researchers have sought to understand the mechanisms by which the chromatin regulatory machinery functions to control higher-ordered genomes. Understanding the function and transcriptional effects induced by various DNA and histone writers, readers, and erasers are a major goal for basic scientists and translational scientists. There are several ways in which scientists have worked towards understanding and controlling these pathways by modulating the activity of a chromatin modifying enzymes, either by inhibiting their catalytic domains [76,77,78], causing their degradation [79], or preventing them from binding [20,80].

By directly binding and inhibiting the catalytic activity of chromatin modifying enzymes, effects on transcription and translation have been inferred. For example, the first two DNMT inhibitors (DNMTi) synthesized, Azacytidine (5-azacytidine) and Decitabine (5-aza-2′-deoxycytidine), are classified as “nucleoside” inhibitors. They mimic the structure of cytidine, are incorporated into the DNA, and form irreversible interactions with DNMTs [81]. Several inhibitors of EZH1 and EZH2 (HMTs that catalyze H3K27 methylation) exist and are classified based on their chemical structure into pyridine-indazole scaffolds (EPZ005687, UNC1999, and GSK343), pyridine-phenyl scaffolds (EPZ006088 and EPZ6438), and pyridine-indole scaffolds (GSK126, CP1-1205, and EI1) [82]. To study and control histone acetylation, a potent and selective p300/CBP inhibitor, termed A-485, was synthesized and shown to bind the catalytic active site of p300, competing with acetyl-CoA [83]. These types of chemicals block function by directly targeting the protein-of-interest.

Another means by which scientists have studied and controlled the chromatin regulatory machinery is through targeted degradation with Proteolysis Targeted Chimeras (PROTACs). These bifunctional molecules bind a protein of interest with one portion of the PROTAC, while the other end binds an E3 ubiquitin ligase protein [84]. Through recruitment of E3 ubiquitin ligase, the protein-of-interest is rapidly degraded. For example, Zengerle et al. developed a PROTAC capable of selectively targeting and degrading BRD4 (a member of the BET family of histone acetylation readers) [79].

Lastly, by blocking the chromatin-based target, the function of specific chromatin marks and enzymes can also be inferred. For example, DNMTis have been designed and synthesized in the “non-nucleoside” category. For instance, Procainamide binds CpG regions of DNA to block DNMT-based activity [80]. These types of chemicals do not bind to the actual DNMT protein, but rather the target, CpG regions of DNA. Another way that chromatin regulation has been studied is through histone replacement. In the approach, the genes that code for histones in *Drosophila* are replaced with histone genes incapable of having certain post-translational modifications. In a seminal paper, McKay et al., mutated the H3K27, H3K36, or H4K20 residue from lysine (K) to Alanine (A), which is incapable of being methylated or acetylated and observed that H3K36 is required for viability and H3K27 is essential for maintenance of cellular identity [20].

In the above examples, global chromatin is being impacted, either through inhibiting or degrading the chromatin modifying machinery or by preventing a DNA or histone modification from being deposited. The disadvantage of chemically inhibiting or degrading chromatin regulatory machinery in the whole cell or organism is that many of the existing chemicals bind off-target proteins. Especially in instances where a conserved domain is being targeting (for example with HDAC class I inhibitors wherein the lysine-binding groove is very similar between all Class I HDACs), specificity is difficult to achieve [85,86,87]. These non-specific binding instances could impact the study’s results or the therapeutic potential. In addition, many of these proteins have multiple substrates, unrelated to chromatin. Downstream effects of the inhibitor could be, at least in part, related to these other substrates [88]. While off-target specificity has been addressed with some of these substrate-blocking methods (CpG binding and histone replacement), the ability to precisely study coordinated pathways with multiple proteins and steps is somewhat limited. In addition, any chromatin-based changes will be epigenome-wide, resulting in direct and indirect changes in gene expression. It is important to recognize that in some disease settings, targeting multiple genes with similar aberrant chromatin modifications is beneficial. When large cohorts of genes are co-repressed by compacted chromatin, targeting multiple genes represents a more efficient solution than targeting one gene at a time [89]. To complement and expand upon previous work with these epigenome-wide approaches, scientists have begun investigating and controlling chromatin environments at a gene-specific level. By targeting defined chromatin modulators to a gene-of-interest, minute mechanistic questions can be answered, and gene-specific transcriptional activity can be controlled.

## 3. Short-Range Locus-Specific Control of Chromatin 

**Figure 1:**
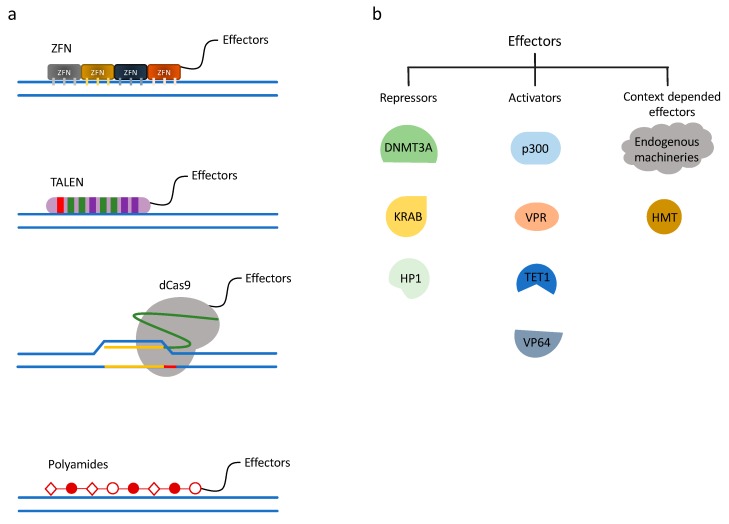
Chromatin engaging technologies and gene regulation systems. (**a**) Chromatin engaging technologies. ZF (zinc finger), TALE (transcription activator-like effector), dCas9, and polyamides are chromatin-engaging techniques used for precise gene targeting, often fused directly to effector proteins. (**b**) Gene regulation systems. Examples of different effectors used in chromatin engaging technologies. By fusing repressive or activating effectors to chromatin engaging platforms, gene regulation can be achieved.

While a lot can be learned through global perturbation of chromatin, site-specific technologies offer an opportunity to examine chromatin regulation in the context of a more physiologic setting without gross changes to the cellular environment. To recruit chromatin effectors to a specific gene, technologies have been developed and utilized to genetically modify a gene-of-interest, insert a Gal4-bind arrays, and recruit Gal4-fused chromatin modifiers [74,90,91,92]. Native to yeast, Gal4 fused to a defined chromatin-modifier would achieve specificity to the mammalian gene of interest. Many other similar proteins with matched DNA binding arrays have also been used (e.g., LexA). The requirement of homologous recombination or other DNA insertion technique to edit genes, in the pre-CRISPR era, made the upfront work for these types of experiments relatively time-consuming and expensive. With the advancements of custom zinc finger nucleases (ZFNs), transcription activator-like effector nucleases (TALENs), and clustered regulatory interspaced short palindromic repeats (CRISPR)/CRISPR-associated (Cas) systems, epigenome editing has been possible without initial modification of mammalian genes [93]. To edit the genome, double-stranded break (DSB) or non-homologues end joining repair pathways are initiated by fusing sequence-specific DNA-binding domains (ZFN or TALEN) to the FokI restriction endonuclease or by creating a sequence-specific single guide RNA (sgRNA) to recruit a Cas endonuclease [94,95,96,97,98]. These techniques were later adapted to serve as a recruitment, rather than DNA-editing, platform and paved the way for targeted chromatin editing, consequently advancing the fields of basic science and translational research. We summarize examples of current technologies to achieve gene-specific control of chromatin in Figure 1.

### 3.1. Zinc Finger Nucleases

ZFNs were the first of these technologies to be developed for editing the mammalian genome (Figure 1a). The Cys_2_-His_2_ zinc-finger domain has been the most common type of zinc-finger used for these editing platforms. Each zinc-finger consists of roughly 30 amino acids (aa) to form a ββα conformation, with a α-helix and the adjacent turn designed to bind 3 bps of DNA. The optimized systems often use six zinc finger domains strung together, targeting 18 bps. This 18 bp length provides specificity within 68 million bases [99]. Zinc fingers have been developed to recognize most of the possible nucleotide triplets, such that pre-designed zinc fingers triples can be linked with molecular cloning techniques in a specific order to target a predefined DNA sequence. If fused to the FokI endonuclease, the zinc fingers are able to induce a DSB in a locus-specific manner. More recently, scientists have used these zinc-finger domains to recruit chromatin modifying machinery to a region of DNA, enabling them to study the resulting chromatin and transcriptional changes [92,100,101]. For example, Snowden et al. targeted the endogenous, mammalian *VEGF-A* locus with a gene-specific zinc finger fused to an HMT. They demonstrated increased H3K9 methylation as well as targeted gene repression [101]. The main disadvantage of the zinc finger technology is that designing and creating zinc fingers is a time-consuming and labor-insensitive process. An alternative way to use this technology is through ordering pre-made constructs to target your gene-of-interest, though they are relatively expensive.

### 3.2. Transcription Activator-Like Effector Nucleases 

Transcription Activator-Like Effector (TALE) proteins are found natively in bacteria and contain a series of 33-35 amino acid repeat domains that recognize a single DNA base pair (Figure 1a). Similar to ZnFs, TALE repeats are strung together to recognize specific DNA sequences [102]. To create a TALE nuclease (TALEN) for the purpose of gene-specific editing, an endonuclease is fused to the TALE. This single-base-pair approach allows for more flexibility in the design than the triple nucleotide design of zinc fingers, however the design and cloning process is more arduous and expensive. Regardless of the upfront efforts, several groups have utilized the TALEs system to study the chromatin-based and transcriptional effects of a given chromatin regulatory protein [92,103,104,105]. For example, Maeder and colleagues demonstrated the ability to actively demethylate human endogenous *RHOXF2* with a TALE-TET1 fusion. Upon recruitment of this fusion protein, CpG methylation levels decreased and transcriptional activity increased [105].

#### 3.2.1. Introduction to dCas9

The clustered regularly interspaced short palindromic repeats (CRISPR) and CRISPR-associated protein (Cas) system provides a technique for mammalian genome editing. Cas9 is the most widely used CRISPR protein for epigenomic engineering. CRISPR RNA (crRNA) and trans-activating crRNA (tracrRNA) recruit Cas9 to a complementary sequence of DNA and sequence-specific nuclease cleavage is introduced. The CRISPR-Cas system was first discovered in prokaryotes as an adaptive immunity to foreign viruses and plasmid DNA. To apply the CRISPR/Cas9 system to mammalian genome manipulation, a single-chain chimeric RNA was created by fusing crRNA and tracrRNA, termed single guide RNA (sgRNA) [106]. This sgRNA contains a complementary sequence to the genome, typically 20 base pairs in length, providing the design capability to target any gene of interest. The protospacer-adjacent motif (PAM) sequence, a unique sequence downstream of the 20-mer, which the Cas9 protein needs for recognizing a target DNA region, is the only required component at the 3′ end of the DNA complementary region [107]. Therefore, compared to ZFN and TALEN, CRISPR/Cas9 can be customized by a 20 base pair synthetic sequence with relatively little molecular cloning. To repurpose Cas9 for epigenome regulation, instead of genome editing, a nuclease deficient Cas9 (dCas9) was developed by introducing mutations at the RuvC1 and HNH nuclease domains (D10A and H841A, respectively) [108]. The dCas9 retains the recruitment characteristics, such that it can bind to a predefined DNA sequence without cutting (Figure 1a). Soon after the dCas9 platform was introduced, an evolving field has grown around dCas9-related protein engineering and technology development to regulate the mammalian epigenome.

#### 3.2.2. Approaches Using Direct Fusions to Activate or Repress Gene Expression

Shortly after Qi et al. published the design of the deactivated Cas9 (dCas9), several labs began to engineer activating protein domains to dCas9 to increase endogenous gene expression. One of the first works published was the dCas9-VP64 fusion from Meader et al. VP64 is a tetrameric repeat of herpes simplex VP16′s minimal activation domain [109]. Meader et al. linked VP64 to the C-terminus of dCas9 and activated expression of human *VEGFA* and *NTF3*. In this work they also created a multiple sgRNA targeting system with which a set of sgRNAs can be co-expressed to target the same gene at several places showing that the activation level of the targeted genes was elevated when compared to using only one sgRNA. A variety of different mammalian genes were activated by other groups by directly fusing activating factors, including VP64 [97,110,111], VP160 (VP16x10) [112], VP192 [113], p65 AD (activation domain) [110], VPR (VP64-Rta-p65) [114,115], Tet1 CD (catalytic domain) [116,117,118], and the p300 core domain [119] (Figure 1b).

Besides exploring activation of a gene by using dCas9 fusion proteins, groups have also demonstrated the possibility of engineered dCas9 proteins to efficiently repress gene expression. Gilbert et al. attached KRAB to dCas9 (Figure 1a,b) and showed repression on a fluorescent reporter gene by 15-fold. Furthermore, RNA-seq analysis indicated high specificity. They next knocked down endogenous gene expression of *CXCR4* and *CD71* in cell lines stably expressing dCas9-KRAB and transduced with sgRNAs [110]. Vojta et al. applied the dCas9 direct fusion technique into DNA methylation and created dCas9-DNMT3A fusion proteins (Figure 1a,b) to specifically increase CpG methylation at a roughly 35 base pair window at the promoter region of the targeted gene locus using multiplexed sgRNAs. They demonstrated that the expression of the targeted endogenous genes was transcriptionally decreased with a concurrent increase in CpG methylation [27]. It is worth mentioning that Qi lab also demonstrated that dCas9 alone can interfere with transcription when the sgRNA targets the protein-coding region by blocking RNA polymerase and transcript elongation [108]. The dCas9-KRAB fusion protein technology has also recently been used in large genome wide screens to map the impact of enhancer elements on gene expression [120].

In addition to the promoter region of genes, different publications have shown the ability to target dCas9 fusions to non-promoter regulatory elements as a means to control the chromatin environment. Hilton et al. expanded the targeting region to core enhancers (CE), distal regulatory regions (DRR), proximal enhancers (PE), and distal enhancers (DE). Both dCas9-p300 core and dCas9-VP64 were used in their experiments targeting *MYOD1*. Based on the activation level of *MYOD1*, sgRNAs targeting the CE, DDR, or promoter region in combination with dCas9-p300 core showed better efficacy than dCas9-VP64. On another gene, the human β-globin locus, by targeting the hypersensitive site 2 (HS2) enhancer region with sgRNAs and dCas9-activator fusion proteins, multiple downstream hemoglobin genes were significantly overexpressed simultaneously [119]. Thakore et al. from the same research group continued working on targeting the same HS2 enhancer with dCas9-KRAB and successfully induced H3K9me^3^ deposition, decreasing chromatin accessibility [121]. Kearns et al. studied and published that cell type-specific regulatory elements can be controlled through recruitment of histone demethylase activity with dCas9-LSD1 [122]. As more attention was drawn to dCas9 technology, a variety of approaches were introduced into this field. Klann et al. developed CRISPR/Cas9-based epigenomic regulatory element screening (CERES) for high-throughput screening of regulatory element activity in their native genomic context. They used dCas9-p300 or dCas9-KRAB constructs and sgRNA libraries to target DNase I hypersensitive sites (regions of open chromatin) surrounding a gene-of-interest. They demonstrated that CERES can identify both known and undiscovered regulatory elements, like HER2 [123]. However, the off-target effects of CRISPR/dCas9 system still need to be addressed by genome-wide readouts.

#### 3.2.3. Indirect dCas9 Recruitment Strategies

After the development of dCas9 direct fusion technologies, researchers developed dCas9-based indirect effectors. The incorporation of SunTag into the dCas9 system has significantly increased the number of epigenetic modulators being recruited. The SunTag is a repeating peptide array, which can recruit a single chain variable fragment (scFv) antibody fusion. Tenenbaum et al. tested the recruitments of 4, 5, 10, or 24 scFv-VP64 fusions and successfully promoted the regulatory effect by increasing the number of antibody fusions [124]. They continued evolving the dCas9-SunTag system with scFv-TET1 [117] and scFv-DNMT3A [125] and observed significant changes in endogenous gene methylation. 

Not only have groups focused on the dCas9 fusion protein engineering, but also on the scaffolding sgRNAs (scRNAs). By engineering the sgRNA with additional stem loops, the recruitment power of the sgRNAs can be increased. Zalatan et al. created three scRNA systems and compared the recruitment efficacy between them. They engineered MS2 stemloops to recruit MS2-capping proteins (MCP), PP7 stemloops to recruit PP7-coat proteins (PCP), and Com stemloops to recruit Com proteins. By fusing MCP, PCP, and Com proteins to VP64, transcriptional activation through stemloop-based recruitment was observed. Furthermore, they developed a two-stemloop version with identical stemloops in the scRNA and the activation was improved [126]. Further optimization of the MS2 system was developed based on the crystal structure of the interaction between dCas9 and sgRNA. Incorporation of the MS2 stemloops in the dCas9-recruiting sgRNA stemloops promoted the recruitment power with MCP-VP64, which was 14-fold more efficient in targeting *ASCL1* than placing the addition loops downstream of the sgRNA stemloops. This improvement likely occurred because either the positioning or the degradation protection of the MS2 protein was improved. In the same study, it was demonstrated that engaging different recruitment strategies (dCas9-VP64 and MCP-P65) was more powerful than using the same activator in both dCas9 and MCP fusions. By utilizing this MS2 stemloop sgRNA, MCP-TET1-CD can activate gene expression by DNA demethylation [127]. With the optimizations of the SunTag system and the scRNA, a side-by-side comparison of dCas9-direct fusions, scRNAs, and SunTag activation efficacy was conducted by Chavez et al. They tested on human, mouse, and fly cell lines, and found that the activation level was effector-, cell line-, and sgRNA-dependent [128].

#### 3.2.4. Inducible Systems

**Figure 2:**
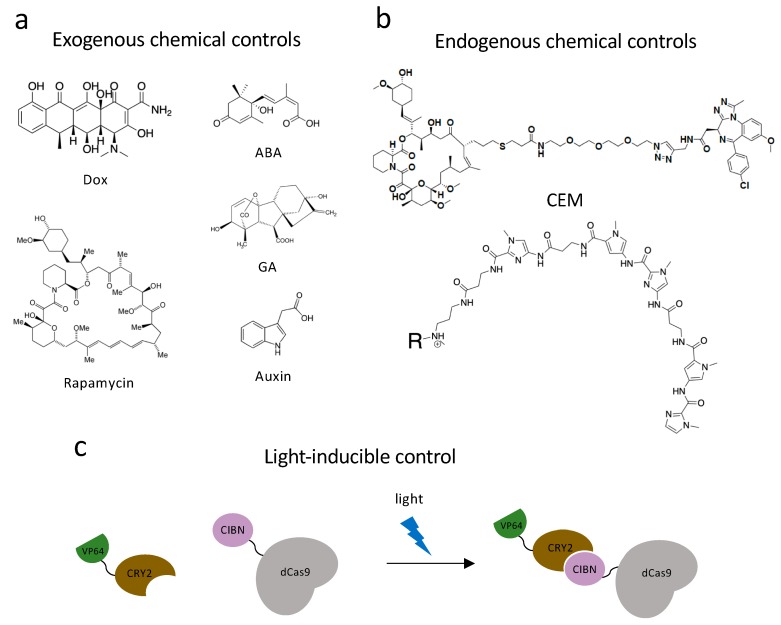
Dynamic control systems. (**a**) Exogenous chemical controls. Dox, rapamycin, ABA (abscisic acid), GA (gibberellic acid) and auxin can interact with DNA-binding systems to recruit effectors to chromatin and achieve dynamic chromatin regulation. (**b**) Endogenous chemical controls. CEMs and Syn-TEFs are synthetic molecules designed to facilitate with chromatin engaging constructs and achieve precise gene regulation by recruiting endogenous protein complexes. (**c**) Light-inducible control technology. Light-inducible heterodimerizing proteins, CRY2 and CIBN, undergo a conformational change in the presence of a specific fluorescent light and bring effectors to chromatin engaging constructs to regulate gene transcriptions.

While researchers were focusing on controlling gene expression by utilizing synthetic devices, several labs began developing chemically responsive gene expression control constructs. By introducing small molecules into biological systems, gene expression can be controlled in a temporal manner. The Conklin lab took advantage of doxycycline (Dox; Figure 2a) and iPSC’s differentiation mechanism and created a doxycycline-inducible dCas9-KRAB repression domain. By introducing Dox into the system, they demonstrated that dCas9-KRAB repression was highly Dox-dependent and reversible [129]. As an attempt at the other direction of chemical control, the Adli lab developed a dCas9 construct that can be turned off by the addition of a plant-based technology called auxin-induced degron. By tethering the degron peptide to dCas9-p300 fusion protein, adding auxin can induce recognition of dCas9 constructs by SCF-E3 ubiquitin ligase through the TIR1 protein. In this technology, roughly 90% of the degron-dCas9-p300 expression can be reduced in a rapid (within 6 h of auxin treatment) and reversible manner [130].

Apart from the two chemically dependent systems mentioned above, there is another approach, chemical inducers of proximity (CIP), being applied to both study and control chromatin biology. This system utilizes a class of bifunctional molecules, which can recruit two protein fusions together and initiate a new biological response. The first CIP was a homodimer of FK506 (FK1012) [131]. FK506 is a calcineurin inhibitor, which can bind FK506-binding-protein (FKBP12) from one side and bind calcineurin on the other. By appending two FK506 molecules into one homodimer, FK1012 can bring together two FKBP proteins. Another FKBP-interacting molecule is rapamycin (Figure 2a), which is a macrolide immunosuppressor. Rapamycin is capable of bringing together FKBP12 and the FKBP12-rapamycin-binding (FRB) domain of the TOR kinase [132]. Three other commonly used CIPs are plant-based molecules, which are advantageous because they are non-toxic while in mammalian systems. Abscisic acid (ABA) is a plant hormone, which functions with the Pyl receptor and phosphatase ABI1 (Figure 2a). Gibberellin (GA) is another plant hormone that induces tethering of GID1 and GAI (Figure 2a). The last common plant-based CIP is indole-3-acetic acid, also named auxin, which was previously used in dCas9 proteasomal degradation (Figure 2a). Auxin can recruit the Cul1 complex to degron-labeled proteins. The discovery and characterization of these CIPs encouraged scientists to develop new techniques and utilize these molecules in diverse ways [132].

With the development of CRISPR/Cas9, many labs have incorporated CIPs and dCas9 fusion proteins to control chromatin structure and gene expression. The Crabtree lab developed a dCas9-based rapamycin, FKBP, and FRB system. By creating ms2-FKBP and FRB-chromatin regulator fusions (e.g., FRB-HP1cs and FRB-BAF), they achieved reversible control of the chromatin environment of endogenous mammalian genes and a change in transcription activities [133]. Zetsche et al. combined rapamycin with a different approach by splitting a dCas9-VP64 fusion into dCas9(N)-FRB and dCas9(C)-FKBP-VP64 [134]. Upon treatment with nanomolar amounts of rapamycin, the C-terminal dCas9 fragment and the N-terminal dCas9 fragment interacted and activated *ASCL* expression at a level comparable to the full-length dCas9-VP64 (57-fold overexpression compared to untreated cells). The Qi lab also developed a highly intricate dCas9 and CIP system. They utilized both ABA and GA to create dCas9-based synthetic gene circuits. They can recruit activating or repressive machinery in a chemically dependent manner by creating dCas9-AB1/Pyl1-fusions and dCas9-GAI/GID1 fusions. The ABA- and GA- inducible recruitments were reversible and orthogonal [135]. Another CIP application was in a split SunTag system whereby the ScFv was fused to FKBP12 and VP64 was fused to a mutated FRB domain (T3089L) [136]. A rapamycin analogue AP21967 that can bind to the mutated FRB selectively over the endogenous TOR protein was developed. This system was used to activate a BFP reporter gene, and 140-fold activation was achieved following treatment with AP21967. For further details of dCas9- and CIP based chromatin regulation please refer to Corson et al. [137] and Stanton et al. [132].

Optogenetic systems have an advantage in precise spatial and temporal controls. Polstein and Gersbach engineered a light-activated dCas9 effector (LACE) system to induce the transcription of endogenous genes by illuminating blue light [138]. They utilized a pair of light-inducible heterodimerizing proteins, CRY2 and CIBN, by fusing CIBN to dCas9 and CRY2 to VP64 (Figure 2c). CIBN-dCas9-CIBN is localized by sgRNA to the targeted gene. In the presence of blue light, CRY2 undergoes a conformational change and heterodimerizes to CIBN, which recruits VP64 to the gene-of-interest and causes transcriptional activation. The Sato group used another pair of light-inducible heterodimerizing domains (positive magnet and negative magnet) and generated photoactivatable dCas9 (padCas9) by splitting dCas9 [139]. They attached the N-terminus of dCas9 to pMag and the C-terminus to nMag and VP64. In the presence of blue light, pMag and nMag will assemble the whole dCas9 protein and targeted endogenous genes will be activated. They successfully induced neuronal differentiation in induced pluripotent stem cells with this approach. Shao et al. incorporated optogenetic far-red light (FRL) to create an activator dCas9 and developed an orthogonal, reversible endogenous gene-activation system (FACE) [140]. FRL has the ability to penetrate deeply into tissues and can activate engineered bacterial photoreceptor BphS, which converts GTP to c-di-GMP. When c-di-GMP concentration is increased, the FRL-dependent transactivator will bind to its chimeric promoter and initiate MS2, P65, and HSF1 transcription. With the expression of MS2 fusions, dCas9 and sgRNA can activate targeted gene expression.

Similar to the split dCas9 protein technology, the Muir lab used another split intein-mediated protein *trans*-splicing strategy (PTS) and designed dCas9-InN (a genetic fusion of dCas9 and the N-terminal fragment of a split intein) and InC-effector. They tested dCas9-InN and InC-VP64 as a direct fusion, which led to transcriptional activation of targeted genes (*IL1RN* and *NTF3*). Next, they expanded the effector into engineered InC-JQ1x4 (small molecule) and InC-UNC3866 (synthetic peptide) and recruited endogenous BRD4 and PRC1 complexes, respectively [141].

Recruiting endogenous chromatin modifying enzymes has a great advantage over exogenous engagement in the simplicity of the technology. With the development of dCas9 approaches to regulate chromatin structure, research groups have expanded the application, utilizing chemical endogenous recruiting warheads. We have developed a bifunctional molecule named chemical epigenetic modifier (CEM), which has two functional components, FK506 and a molecule interacting with cellular epigenetic machinery [142] (Figure 2b). We incorporated dCas9 with MS2 sgRNA stemloops and MCP-FKBPx2 fusion and determined it to be the most effective dCas9 recruiting machinery after screening a series of other recruitment systems including dCas9-FKBP and SunTag system. Different recruiting warheads were tested to target endogenous genes (*CXCR4*, *MYOD1*, etc.) in multiple cell lines, and CEM87, which is composed of FK506 and iBet762 (a BRD inhibitor), was determined to be the best-in-class CEM. With different concentrations of CEM treatment, graded activation of targeted genes can be achieved. CEM can also provide reversible gene activation by washing out the molecule. To determine the off-target effects, ChIP-sequencing (ChIP-seq) and RNA-sequencing (RNA-seq) were conducted. With expression of the targeting sgRNA and addition of CEM87, no off-target effects were observed, which is indicative of the specificity of CEM technology.

### 3.3. Synthetic Transcription Factors 

Transcription factors (TFs) are diverse signaling proteins that play important roles in gene expression and cell differentiation in a dynamic manner. During cellular development, transcription factors interpret signals and initiate programmed transcriptional changes. Artificial transcription factors (ATFs) have been designed and customized to reprogram somatic cells into induced pluripotent stem cells (iPSCs) [143]. Repurposing ATFs as a toolkit to regulate transcription provides another category of gene-specific targeting and regulatory technology. ATFs are composed of a DNA Binding Domain (DBD), an Interaction Domain (ID), and an Effector Domain (ED). TALEs, ZnFs, CRISPR-Cas9, and polyamides can serve as the DBD. Pyrrole- and imidazole-based polyamides are a class of synthetic molecules developed by Ansari and colleagues, designed to be sequence-specific synthetic transcription elongation factors (Syn-TEFs), which are programmable DNA-binding ligands that target desired genomic loci and engage the transcription elongation machinery [144] (Figure 2c). The programmed polyamides were designed to bind at the frataxin (*FXN*) gene and were conjugated to JQ1 (BRD4 ligands). By recruiting BRD4 to *FXN*, JQ1 can engage the transcription elongation machinery P-TEFb and facilitate transcription elongation. Activation of *FXN* was observed in lymphoblastoid cell lines, and no off-target effects were seen with RNA-seq and ChIP-seq. With treatment of Syn-TEF to overexpress *FXN*, they induced iPSCs to differentiate into cardiomyocytes that expressed cardiac-specific markers and were observed to beat rhythmically in culture. Finally, they examined the utility of Syn-TEF in vivo by establishing an immunocompromised mouse model bearing a luciferase reporter fused with *FXN*, and upon Syn-TEF treatment luciferase expression was stimulated. More ATF technologies can be found in the review written by Heiderscheit et al. [145].

## 4. Translational Implications

Discoveries over the past few decades have demonstrated the importance of chromatin regulatory pathways and have implicated their dysregulation in several human diseases (Table 1). Technological advances and new ways of thinking have paved the way for understanding how these processes are dysregulated in diseased settings, what the downstream transcriptional consequences are, and how best to correct these changes. Epigenetic-based clinical trials are ongoing and dozens of epigenetic-based drugs have been approved, yet more specific and safer approaches are needed.

With the understanding that changes in chromatin can contribute to disease initiation, growth, and/or therapeutic resistance, small molecules have been developed to study epigenome modulators and regulate their activity [194]. The idea of using epigenomic agents in combination with the standard-of-care therapy regiment has been around for over 40 years [195]. As previously discussed, targeting chromatin regulators in a cell-wide manner can result in off-target effects. Chromatin modifying enzymes can have non-histone substrates. The inhibition of an acetyl transferase, for example, could result in undesired effects. In addition, dysregulation of these pathways impacts the expression of dozens to thousands of genes, while only a small percentage of these gene expression changes are contributing to the diseased phenotype. Lastly, many of these inhibitors have limited target specificity, resulting in inhibition of other proteins. All of these whole-cell changes and the lack of specificity results in less effective drugs and increased toxicity in patients. For example, high levels of toxicity have been observed in patients treated with HDACi [196], likely due to poor bioavailability and/or off-target binding effects [195,197]. It has been demonstrated that the compounds also inhibit methylation and phosphorylation of non-histone substrates [198]. A trial using Vorinostat in combination with a chemotherapy (docetaxel) was terminated due to toxicities including neutropenia, peripheral neuropathy, and gastrointestinal bleeding [199]. Additional patient toxicities include fatigue, nausea, vomiting, thrombocytopenia, and weight loss [200]. While improvements in development of potent, targeted drugs have been made, the ability to control the chromatin environment and the expression of specific genes and/or target exposure to exclusively cancer cells are areas being actively pursued in research.

In some cases, groups have been able to specify a tumor suppressor or oncogene for a given disease, in which the chromatin environment is altered at the gene locus, thereby affecting the transcriptional activity (Table 2). These examples represent ideal targets for gene-specific control of chromatin environments. In addition to manipulating the chromatin environment at a specific gene, this level of control enables scientists to model mislocalization of chromatin machinery and specific changes in histone or DNA modifications, to then determine the direct downstream recruitment and transcriptional effects.

While gene-specific control of chromatin is not currently possible in the clinical setting, several recent advances have brought the field much closer to this being a reality. CRISPR-Cas9-based therapies have already been done in patients ex vivo [214], the dCas9 system has been demonstrated as a therapeutic approach in murine models [215], Syn-TEF has advanced into in vivo studies in immunocompromised mouse models, and delivery systems such as AAV [216] and whole-protein-deliveries [217] are also being optimized. Simultaneously, advancements have been made in decreasing the size of the CRISPR system, providing greater efficiency in delivery systems such as AAV. This has been through the discovery of smaller versions Cas9 (*Staphylococcus aureus* dCas9) [218,219], smaller Cas9-like CRISPR systems (deactivated CasX [220] and deactivated Cpf1 [221,222]), CRE-inducible CRISPR [223], molecular engineering of artificial split Cas9 genes [136], and *sa*-Cas9 proteins lacking non-essential domains (mini-Cas9 [224]). The smaller size of these emerging Cas variants will greatly contribute to making gene-specific control of chromatin a clinical reality. The stability of epigenomic modifications must also be addressed. Unless the gene-specific chromatin modifier is constituently expressed in the target tissue many publications have reported reversions back to the initial chromatin state before synthetic perturbation. For instance Kungulovski et al. found that in ovarian cancer cells that changes made by ZF-DNMT or ZF-HKMT fusion proteins were not stably maintained even 10 days after adenoviral treatment [225].

## 5. Conclusions

Development of new ways to tether proteins and proteins associated with RNA to chromatin has opened up the ability to modulate specific endogenous genes by an assortment of chromatin modifying activities. Using gene-specific targeting technologies in conjunction with chromatin regulators has allowed for directed epigenomic engineering. Furthermore, bringing temporal and spatial control with these technologies has allowed finer governing of chromatin state at specific times and in subsets of cell populations. These technologies have been used to study the mechanisms of chromatin regulation in new ways and across a diverse set of genes. Modern research is also examining the ability of these approaches to be used to treat human diseases, yet most of this is still in the preclinical setting. The overexpression of synthetic effectors is required in most of the technologies summarized in this review, having undesirable impacts to the epigenome. While epigenomic engineering has been empowered by technology, it is likely to be driven further by techniques that are yet to be developed. Expect the future to hold a wider assortment of chromatin regulatory activities to be tethered to chromatin as only a subset of all the wide array of chromatin regulatory enzymes have been tested by these approaches. Additionally, new methods to anchor chromatin-modifying enzymes to target genes will lead to even more effective and specific chromatin modulation than current techniques.

## Figures and Tables

**Table 1:** Histone and DNA writers, readers, and erasers modified in diseases.

Mutated Writers, Readers, and Erasers	Alteration	Disease(s)
DNMT3A	Missense, Frameshift, Nonsense, Splice site	AML [146], MDS [147]
TET1	Translocation	AML [148]
TET2	Missense, Nonsense, Frameshift	MPN [149], AML [150], MDS [151], CMML [152]
CBP/KAT3A	Translocation, Nonsense, Missense, Frameshift	AML [153,154], DLBCL [155], MDS [156], RTS [157]
P300/KAT3B	Translocation, Nonsense, Missense, Frameshift	CML [158], Pancreatic [159], Colorectal [159], Breast [159], DLBCL [155], AML [160]
MOZ/KAT6A	Translocations	AML [160,161]
MORF/KAT6B/MYST4	Translocations	AML [153], Uterine Leiomyomata [162]
BRD2	Unknown	ALL [163]
BRD3	Translocation, Missense	Midline Carcinoma [164], Lung [165]
BRD4	Translocation	Midline Carcinoma [166]
TRIM33	Translocation	Lung [167]
KMT2A/MLL1	Partial Tandem Duplication, Translocation	AML [168], ALL [169]
KMT2B/MLL2	Nonsense, Frameshift, Missense	Medulloblastoma [170], Breast [171], Renal [172], DLBCL [173], Prostate [174], FL [173], Lung [175]
KMT2X/MLL3	Nonsense	Medulloblastoma [170], Breast [171]
KMT3A/SETD2	Nonsense, Frameshift, Missense	Renal [172], Breast [171]
KMT3B/NSD1	Translocation	AML [176]
NSD2	Translocation, Missense	Multiple Myeloma [177], ALL [178]
NSD3	Translocation	AML [179]
KMT6/EZH2	Missense	DLBCL [180], MPN [181], MDS [182]
KDM5A/JARID1A	Translocation	AML [183]
KDM5C/JARID1C	Nonsense, Frameshift, Splice site	Renal [172]
KDM6A/UTX	Deletion, Nonsense, Frameshift, Splice site	AML [184], Renal [184], Esophageal [184], Multiple Myeloma [184], CML [184]
PHF6	Deletion, Missense	T-ALL [185], AML [186]
BRD8	Missense, Nonsense	Liver [187]
DNMT1	Nonsense, Missense	Colon [188]
HDAC2	Frameshift	Colon [189]
HDAC9	Missense	Prostate [190]
PRDM9	Nonsense, Missense	Head and Neck [191]
SETD2	Frameshift, Nonsense, Splicing site	Glioblastoma [192], Renal [193]
SETD1A	Nonsense	Breast [171]

**Table 2:** Genes with modified chromatin environments in a diseased context.

Gene	Chromatin Change	Disease (s)	Reference
TP53	Promoter hypermethylation	Glioblastoma	[201]
CDKN2A	Promoter hypermethylation	Burkitt’s lymphoma	[202]
	Increased H3K9me^2^	Liver cancer	[203]
TSSC3	Promoter hypermethylation	Osteosarcoma	[204]
FSHD	Decreased CpG methylation	FSHD	[205]
miR-181c	Promoter hypermethylation	Glioblastoma	[206]
Sat2	Loss of H3K4me^3^	Leukemia	[207]
p21	Decreased H3ac and H4ac	Bladder cancer	[208]
IL6	H3K9me^2^	Type 1 diabetes	[209]
TAL1	Decreased H3K27me^3^/Increased H3K27ac	T-ALL	[210]
TMPRSS4	Decreased DNA methylation	Lung cancer	[211]
RASSF1A	De Novo DNA methylation	Breast cancer	[212]
STAT1/MyD88	Increased H3K9ac	Type 1 diabetes	[213]
